# A Case Report of Pemphigus Vulgaris Initially Misdiagnosed as Tinea Capitis: Infectious Consequences of Diagnostic Delay in a Patient Treated With Rituximab

**DOI:** 10.7759/cureus.87662

**Published:** 2025-07-10

**Authors:** José C González-Rodríguez, Maria Cristofori, José A Antunez Oliva, Emmanuel E Cortés-Marín

**Affiliations:** 1 Internal Medicine, Universidad de Costa Rica, San José, CRI; 2 General Practice, Universidad de Ciencias Médicas (UCIMED), San José, CRI

**Keywords:** delayed diagnosis, intravenous immunoglobulin, pemphigus vulgaris, rituximab, tinea capitis

## Abstract

Pemphigus vulgaris is a rare and potentially life-threatening autoimmune blistering disorder affecting the skin and mucous membranes. Prompt diagnosis and appropriate treatment are crucial in preventing severe complications. We report the case of a 53-year-old woman with severe pemphigus vulgaris who was misdiagnosed as having tinea capitis for approximately four months. During this time, she received multiple courses of systemic antifungals and antibiotics without clinical improvement, resulting in the progressive dissemination of lesions, including mucosal and ocular involvement. The correct diagnosis was ultimately established through skin biopsy, and immunosuppressive therapy with prednisone and rituximab was initiated. During immunosuppression, the patient developed severe infections due to *Pseudomonas aeruginosa*, extended-spectrum beta-lactamase (ESBL)-producing *Escherichia coli*, and severe acute respiratory syndrome coronavirus 2 (SARS-CoV-2), necessitating the temporary suspension of rituximab and the introduction of intravenous immunoglobulin as a bridging strategy. The patient experienced favorable clinical evolution, with more than 80% reepithelialization and the resolution of infectious complications. This case underscores the importance of considering autoimmune diseases in treatment-refractory scalp dermatoses, avoiding prolonged empirical antimicrobial use, and employing a multidisciplinary approach in immunosuppressed patients at high risk of infection.

## Introduction

Pemphigus vulgaris is an autoimmune blistering disease that affects the skin and mucous membranes, histologically characterized by suprabasal acantholysis secondary to autoantibodies against desmogleins [1]. Although scalp involvement is common in advanced stages, when lesions begin in this location and manifest as crusted erosions or patchy alopecia, they can mimic common infectious entities such as tinea capitis, complicating the early recognition of the condition [2-4].

This case describes a patient with severe pemphigus vulgaris who was initially treated for several weeks as having tinea capitis, receiving multiple courses of systemic antifungals and antibiotics. The definitive diagnosis was established late, at a stage of extensive disease with mucosal and ocular involvement. After initiating immunosuppressive therapy with rituximab and corticosteroids, the patient developed serious infections, namely, *Pseudomonas aeruginosa* bacteremia, urinary tract infection caused by extended-spectrum beta-lactamase (ESBL)-producing *Escherichia coli*, and severe acute respiratory syndrome coronavirus 2 (SARS-CoV-2), which required adjustments to the therapeutic approach.

This case is significant because it illustrates how delayed diagnosis in blistering disorders may not only exacerbate clinical severity but also predispose patients to unnecessary antimicrobial exposure, increasing the risk of colonization with multidrug-resistant organisms [5]. When potent immunosuppression is required, such conditions markedly elevate the risk of infection and complicate therapeutic decision-making [6]. This experience highlights the importance of considering autoimmune diseases in treatment-refractory scalp dermatoses [7] and underscores the need for close monitoring in immunosuppressed patients with prolonged antimicrobial exposure.

## Case presentation

**Figure 1:**
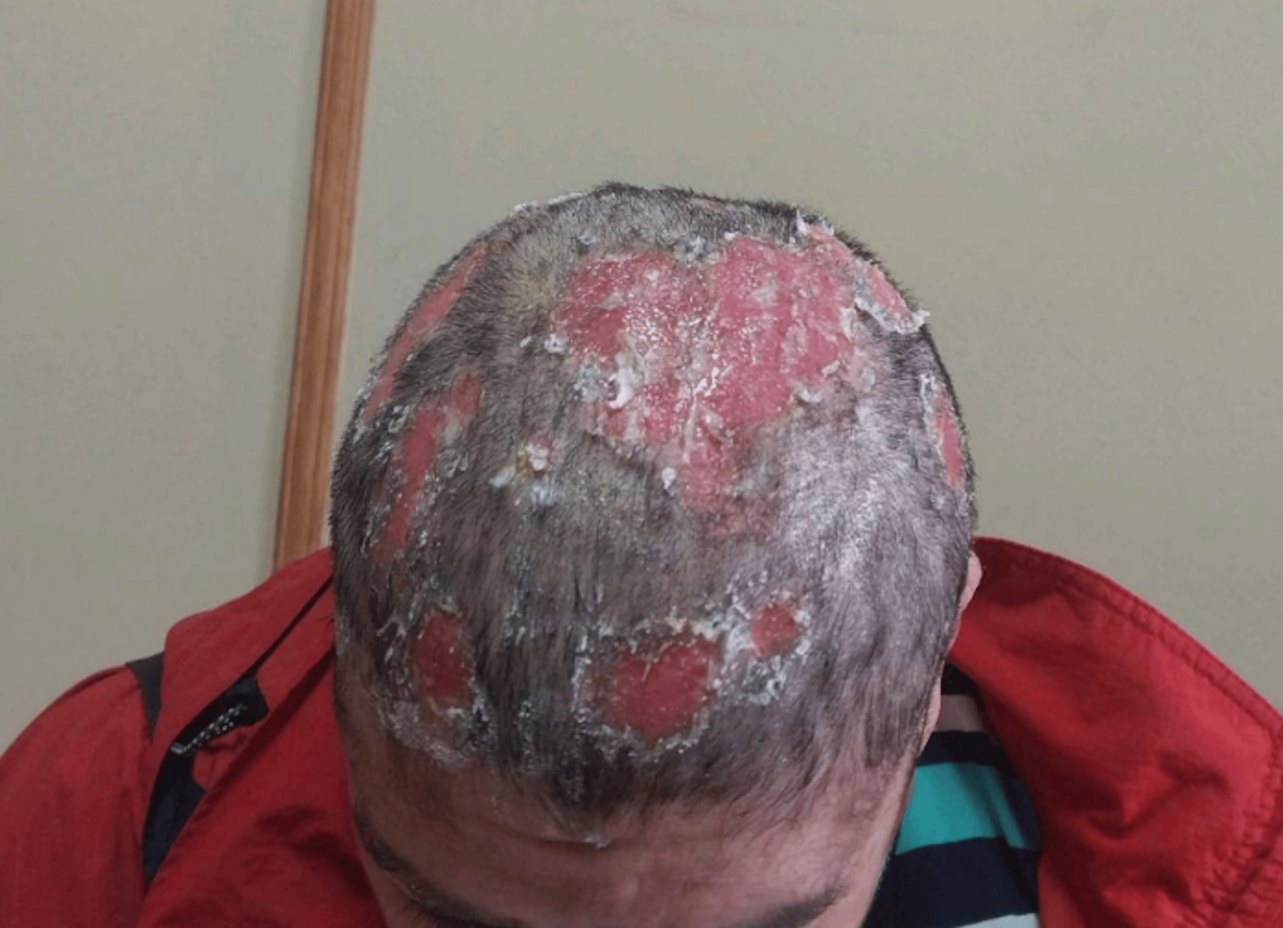
Initial clinical presentation on the scalp Scalp involvement prior to diagnosis, showing exclusive cutaneous lesions: extensive erosive and crusted plaques with focal alopecia affecting the scalp, initially misinterpreted as tinea capitis.

A 53-year-old female patient with a past medical history of hypertension, type 2 diabetes mellitus, and hypothyroidism initially presented to primary care with erosive lesions on the scalp, covered by serohematic crusts with a foul odor and associated alopecia in the affected areas, as shown in Figure 1.

**Figure 2:**
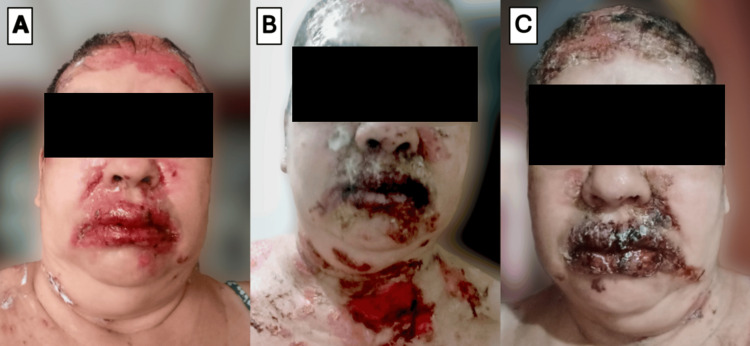
Evolution of facial and perioral lesions in untreated pemphigus vulgaris Serial clinical photographs of the patient before the start of adequate therapy. (A) Appearance at the time of diagnosis, showing extensive erosions and early hemorrhagic crusts on the forehead and perioral regions. (B-C) Subsequent photographs showing the natural progression of the disease before effective treatment was initiated. The evolution demonstrates the formation of widespread, thick, adherent hemorrhagic crusts over the initial erosions, which is characteristic of untreated pemphigus vulgaris, as fragile bullae rupture and the raw surfaces become secondarily crusted. The photographs were taken by the patient.

**Figure 3:**
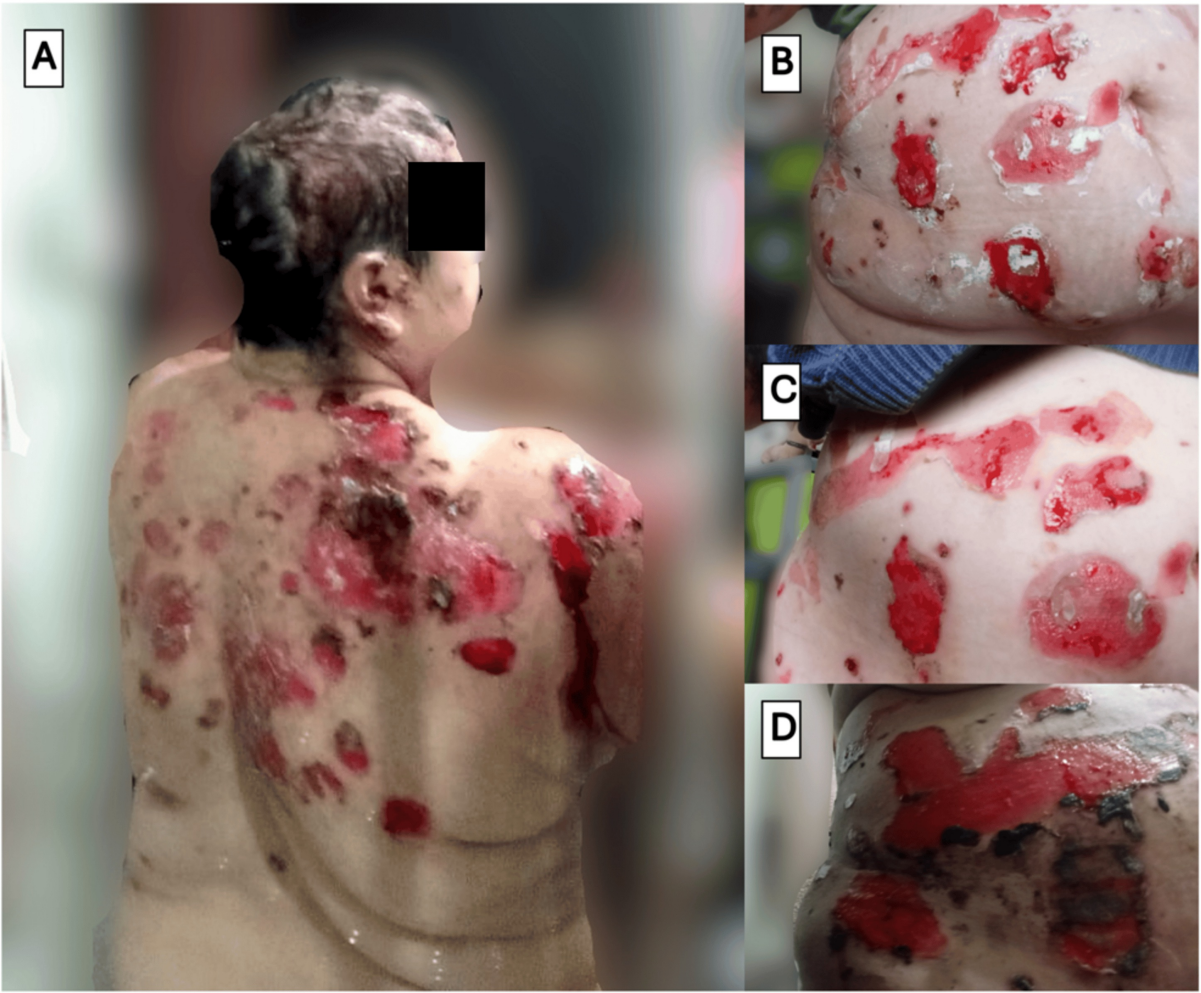
Widespread truncal and back manifestations of pemphigus vulgaris Clinical photographs demonstrating the extent and evolution of cutaneous involvement on the patient's trunk and back prior to effective therapy. (A) Numerous large, irregular erosions with hemorrhagic crusts distributed across the upper back and shoulders. (B-D) Serial photographs of the same abdominal region at different moments, documenting the natural history of the lesions. The progression begins with multiple, moist, well-demarcated erosions (B, C) that subsequently evolve to become covered by large, confluent hemorrhagic crusts (D). The photographs were taken by the patient.

The condition was managed as tinea capitis with secondary bacterial superinfection. Topical treatment with miconazole and fusidic acid was initiated, followed by oral itraconazole and multiple courses of systemic antibiotics, including cephalexin, doxycycline, and trimethoprim-sulfamethoxazole, based on a superficial culture that grew *Staphylococcus aureus*. Despite frequent wound care and more than two months of therapy, there was no clinical improvement. On the contrary, the lesions progressed extensively. The involvement of the face, neck, and oral and ocular mucosae is illustrated in Figure 2, while the widespread lesions on the trunk are depicted in Figure 3.

**Figure 4:**
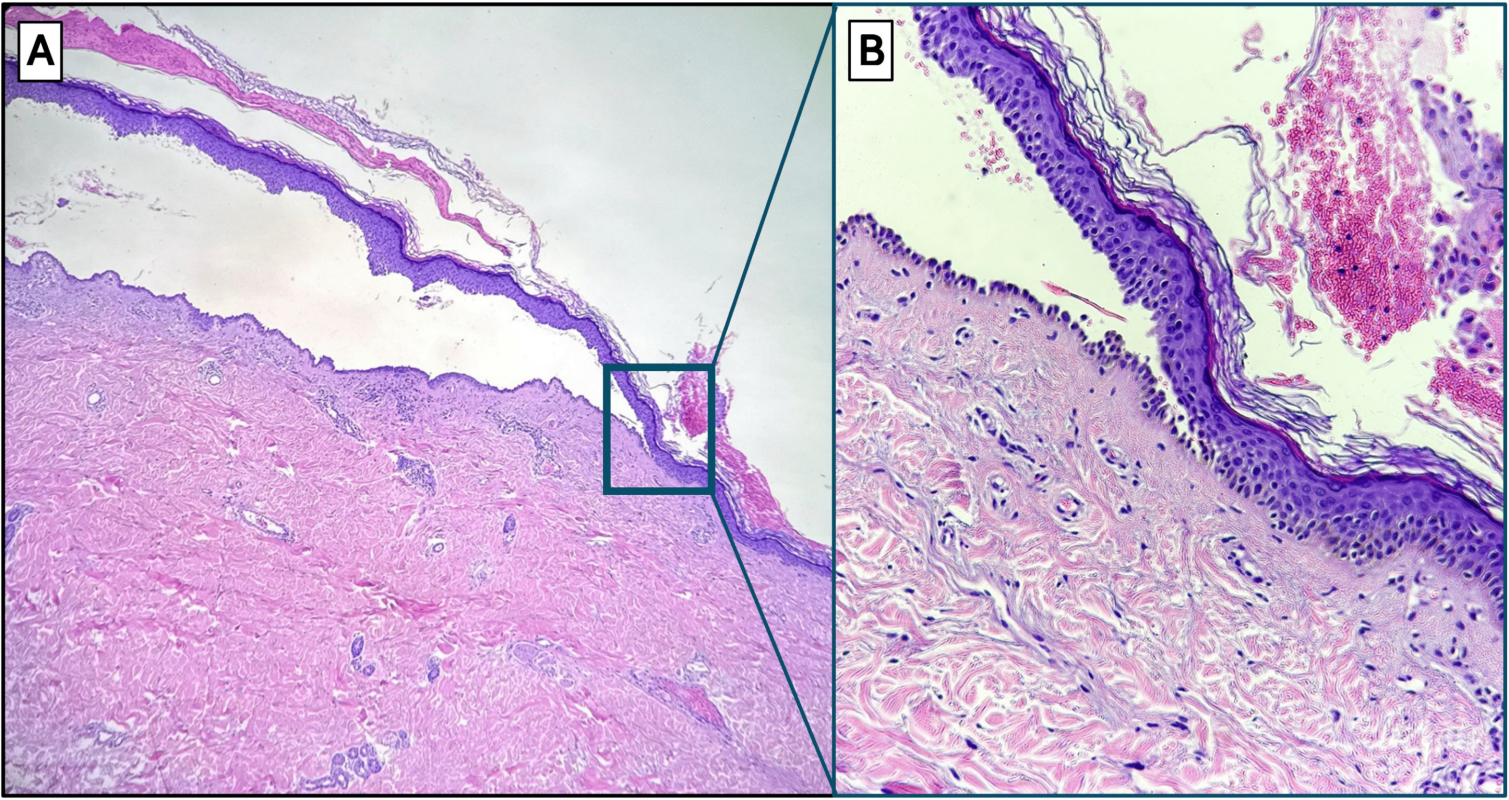
Suprabasal acantholytic intraepidermal blister and dermal inflammation in skin biopsy (hematoxylin and eosin stain) (A) Low-power view (original magnification ×4) of a skin biopsy showing an intraepidermal suprabasal blister with acantholytic features, characteristic of pemphigus vulgaris. The blister cavity is located above the basal layer, which remains attached to the dermis ("row of tombstones" appearance). (B) High-power magnification (original magnification ×10) highlighting acantholytic keratinocytes within the blister cavity and dense perivascular lymphohistiocytic inflammation in the superficial dermis.

She was evaluated by the dermatology service, where a skin biopsy confirmed the diagnosis of pemphigus vulgaris approximately four months after the onset of symptoms. Histopathological analysis of a sample taken from the right arm revealed an intraepidermal suprabasal blister with acantholytic keratinocytes and a dense perivascular lymphohistiocytic infiltrate in the superficial dermis, as shown in Figure 4. Although direct immunofluorescence is considered the gold standard for diagnosing pemphigus vulgaris, it was not performed in this case due to the unavailability of the test at the pathology laboratory.

Given that approximately 50% of the total skin surface was affected, she was referred for multidisciplinary evaluation and hospital admission. Upon admission, she presented with multiple eroded flaccid bullae involving the scalp, oral mucosa, face, trunk, and arms. Involvement of the anterior lamella of both eyelids was also documented. The Pemphigus Disease Area Index (PDAI) activity score was calculated at 122 points, consisting of 75 points for cutaneous involvement, 10 points for the scalp, and 37 points for mucosal lesions. The PDAI is a validated scoring system used to quantify disease severity and guide treatment response in pemphigus, with scores above 45 indicating severe disease [1].

In the absence of active infection, oral prednisone at a dose of 1 mg/kg/day was initiated, along with the first dose of rituximab at a dose of 1 g in intravenous infusion, following the rheumatoid arthritis (RA) protocol. This regimen was used in this case due to local practice patterns and the high cost of the medication [1,6].

On the 10th day of immunosuppressive therapy, the patient developed upper respiratory symptoms and tested positive for SARS-CoV-2. Two days later, a marked elevation in procalcitonin led to further evaluation, revealing a central line-associated bloodstream infection (CLABSI) due to *Pseudomonas aeruginosa*. Additionally, she developed a non-catheter-associated urinary tract infection caused by *Pseudomonas aeruginosa* and ESBL-producing *Escherichia coli*. Targeted intravenous antimicrobial therapy with ceftazidime and amikacin was initiated.

Due to the infectious complications, the second dose of rituximab (originally scheduled for day +15 after the first infusion) was postponed, and intravenous immunoglobulin (IVIG) was administered as a bridging strategy, at a total dose of 160 grams over five days.

**Figure 5:**
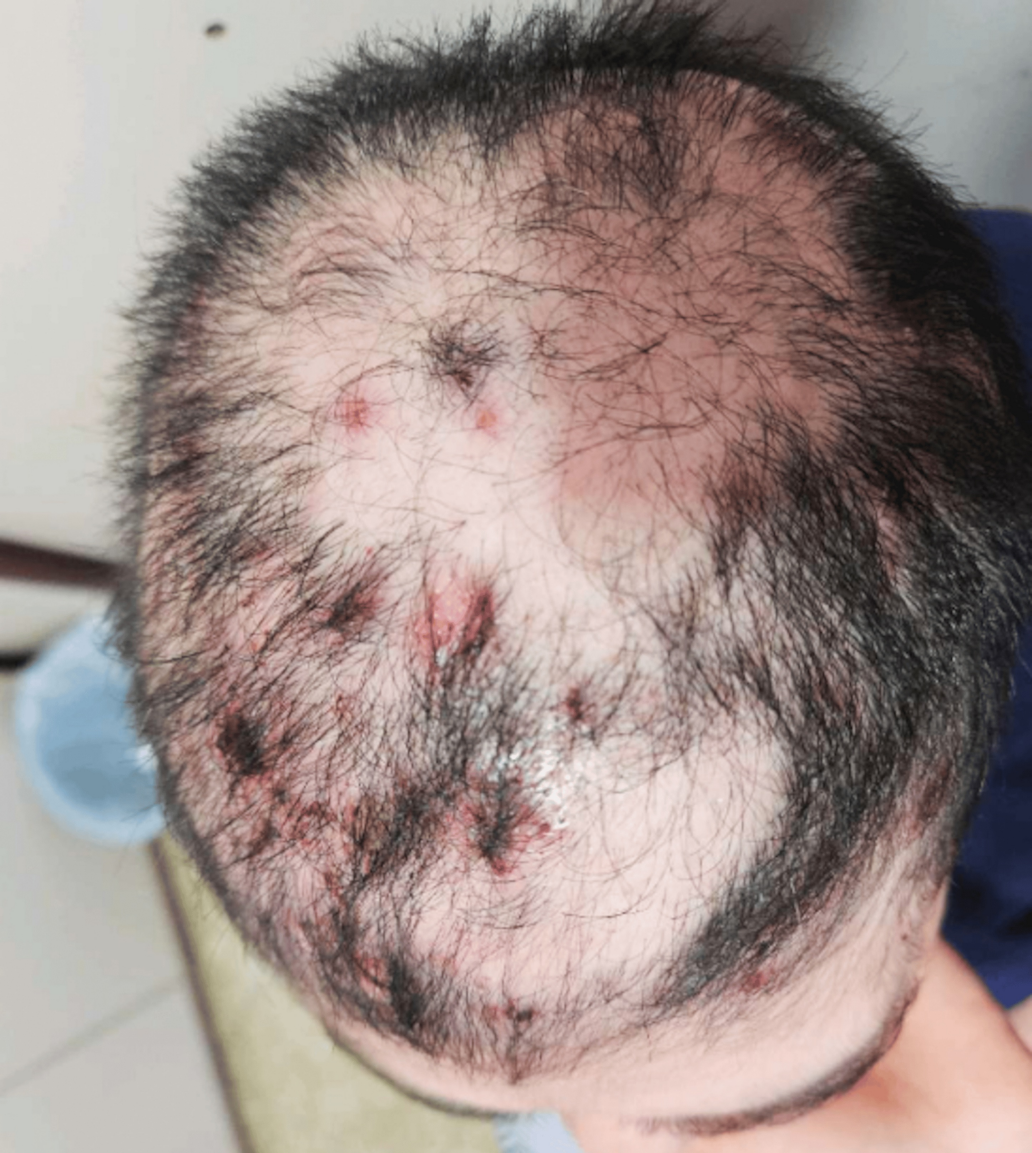
Scalp lesion evolution at day +22 Clinical improvement following the initiation of rituximab and oral corticosteroids: reepithelialization and reduction in lesion severity observed on the scalp 22 days after treatment initiation.

The patient showed a favorable clinical course, with reepithelialization of more than 80% of the cutaneous lesions, sustained reduction in acute-phase reactants, and clinical resolution of the infectious processes. Figure 5 illustrates the improvement in the scalp skin. She completed seven days of antibiotic therapy, remained asymptomatic for COVID-19, and was kept in isolation for 21 days in accordance with institutional protocol. After the resolution of the infectious conditions, the second dose of rituximab was administered without complications.

At the time of discharge, the patient was in good general condition and afebrile, with no new lesions and stable hemodynamic, renal, and hepatic function. A multidisciplinary outpatient follow-up was planned.

## Discussion

Pemphigus vulgaris is a severe autoimmune disease in which timely diagnosis significantly improves prognosis. Although it commonly presents with generalized mucocutaneous lesions and may involve the scalp during disease progression, up to 15% of cases may initially present with scalp lesions, mimicking infectious dermatoses such as tinea capitis, thereby delaying clinical recognition [8].

In this case, the initial presentation on the scalp, characterized by crusted erosions and focal alopecia, led to a misdiagnosis of tinea capitis with secondary bacterial infection, a diagnostic pitfall documented in the current literature [2,4,7]. The lack of improvement after multiple courses of antifungal and antimicrobial therapy should raise suspicion for alternative diagnoses. Early confirmation through skin biopsy and direct immunofluorescence is essential to prevent clinical progression [9-11].

The differential diagnosis of erosive and crusted scalp lesions is broad, encompassing both infectious and non-infectious etiologies. Apart from tinea capitis, other infectious mimics include impetigo, folliculitis decalvans, and actinomycosis. Among inflammatory and autoimmune conditions, erosive pustular dermatosis of the scalp (EPDS) is a key consideration, particularly in elderly patients with sun-damaged skin or prior trauma. Other autoimmune conditions, such as discoid lupus erythematosus, lichen planopilaris, and cicatricial pemphigoid, may also manifest with scarring alopecia and chronic erosive lesions. Additionally, cutaneous T-cell lymphoma and skin metastases should be considered in persistent, atypical, or therapy-refractory presentations [1,7].

Although pemphigus vulgaris primarily affects the skin and the oral and nasal mucosa, ocular involvement, while uncommon, can occur in severe forms of the disease. It may present as erosive conjunctivitis, keratitis, or synechiae [12]. In this patient, involvement of the anterior lamella of the eyelid was documented, an uncommon finding, but one that has been described in the context of extensive disease. Recognition of ocular manifestations is critical, as they may lead to photophobia, persistent ocular pain, and a risk of long-term visual sequelae if not promptly managed [13,14].

Treatment with rituximab in combination with corticosteroids has become the first-line therapy for pemphigus vulgaris. Recent observational studies report complete remission rates exceeding 85% within six months of treatment initiation, using various regimens [15,16]. Moreover, disease-free persistence has been documented in 86% of patients for up to 34 months [17].

However, profound immunosuppression carries a high risk of severe infections, particularly in patients with multiple risk factors and prior prolonged antimicrobial exposure [17,18]. In this patient, bacteremia due to *Pseudomonas aeruginosa* and a urinary tract infection caused by ESBL-producing *Escherichia coli *were documented, along with viral infection by SARS-CoV-2, reflecting the synergistic impact of biotherapy, corticosteroids, and antibiotic-induced dysbiosis [19].

The use of IVIG as a bridging therapy has been described in recent case series as an effective strategy to stabilize disease activity while active infections are being controlled, with an acceptable safety profile. Beyond its role as a temporary bridge to other immunosuppressive therapies, IVIG also possesses immunomodulatory properties, making it a potentially valuable therapeutic option in patients with pemphigus vulgaris complicated by severe or recurrent infections [20].

Furthermore, the infectious complications that arose during immunosuppression point to the need for prophylactic strategies in high-risk patients. These may include *Pneumocystis jirovecii* pneumonia (PJP) prophylaxis with trimethoprim-sulfamethoxazole in patients receiving prolonged corticosteroids, strict central line care protocols to reduce CLABSI risk, and earlier consideration of IVIG as a steroid-sparing or bridging agent in patients with a history of antimicrobial exposure or colonization with resistant organisms [1,18,19].

Finally, this case underscores the importance of maintaining a high index of diagnostic suspicion in treatment-refractory dermatoses, particularly when the clinical course does not follow the expected pattern of infectious diseases. It highlights not only the therapeutic complexity of severe pemphigus vulgaris but also the critical importance of early multidisciplinary involvement, including dermatology, to avoid diagnostic delays. In this patient, dermatologic evaluation and biopsy were not obtained until approximately four months after symptom onset, underscoring the need for earlier specialist referral in cases of treatment-refractory dermatoses. A multidisciplinary approach, encompassing dermatology, internal medicine, infectious diseases, and pharmacy, is essential not only for managing immunosuppressive therapy but also for recognizing atypical presentations and minimizing complications.

The case also reflects the challenges faced in settings without access to dermatologic intensive care units, where specialized support for patients with severe blistering diseases is limited. In such contexts, early referral and strong interdisciplinary collaboration become even more critical.

## Conclusions

This case highlights the clinical implications of a delayed diagnosis of pemphigus vulgaris with an atypical presentation on the scalp, mimicking an infectious dermatosis. The progression to extensive disease with mucosal and ocular involvement, along with the development of severe infections during immunosuppressive therapy, reflects the therapeutic challenges in this context.

This experience emphasizes the need to consider autoimmune blistering diseases in treatment-refractory dermatoses, to limit prolonged empirical antimicrobial exposure, and to implement a multidisciplinary approach that balances immunologic control with the prevention and timely treatment of infections.

A systematic evaluation from the outset can help prevent complications and optimize both functional and survival outcomes for the patient.
